# Accounting for extinction dynamics unifies the geological and biological histories of Indo-Australian Archipelago

**DOI:** 10.1098/rspb.2024.0966

**Published:** 2024-09-25

**Authors:** Leonel Herrera-Alsina, Lesley T. Lancaster, Adam C. Algar, Greta Bocedi, Alexander S. T. Papadopulos, Cecile Gubry-Rangin, Owen G. Osborne, Poppy Mynard, Simon Creer, Rafael Villegas-Patraca, I. Made Sudiana, Fahri Fahri, Pungki Lupiyaningdyah, Meis Nangoy, Djoko T. Iskandar, Berry Juliandi, David F. R. P. Burslem, Justin M. J. Travis

**Affiliations:** ^1^ School of Biological Sciences, University of Aberdeen, Aberdeen AB24 2TZ, UK; ^2^ Deparment of Biology, Lakehead University, Thunder Bay, Ontario, Canada P7B 5E1; ^3^ School of Natural Sciences, Bangor University, Bangor LL57 2DG, UK; ^4^ Departamento de Biología Evolutiva, Instituto de Ecología, AC (INECOL), Xalapa, Veracruz 91073, Mexico; ^5^ Research Center for Biology, Indonesian Institute of Sciences, Jakarta, Indonesia; ^6^ Department of Biology, Tadulako University, Palu, Indonesia; ^7^ Zoology Division, Museum Zoologicum Bogoriense, Research Center for Biology, Indonesian Institute of Sciences (LIPI), Cibinong, Indonesia; ^8^ Faculty of Animal Husbandry, Sam Ratulangi University, Kampus Bahu Street, Manado 95115, Indonesia; ^9^ Department of Biology, FMIPA Institut Teknologi Bandung 10 Jalan Ganesa, Bandung 40132, Indonesia; ^10^ Department of Biology, Faculty of Mathematics and Natural Sciences, IPB University, Bogor 16680, Indonesia

**Keywords:** extinction dynamics, biogeography, interdisciplinarity, vicariance, dispersal

## Abstract

Biogeographical reconstructions of the Indo-Australian Archipelago (IAA) have suggested a recent spread across the Sunda and Sahul shelves of lineages with diverse origins, which appears to be congruent with a geological history of recent tectonic uplift in the region. However, this scenario is challenged by new geological evidence suggesting that the Sunda shelf was never submerged prior to the Pliocene, casting doubt on the interpretation of recent uplift and the correspondence of evidence from biogeography and geology. A mismatch between geological and biogeographical data may occur if analyses ignore the dynamics of extinct lineages, because this may add uncertainty to the timing and origin of clades in biogeographical reconstructions. We revisit the historical biogeography of multiple IAA taxa and explicitly allow for the possibility of lineage extinction. In contrast to models assuming zero extinction, we find that all of these clades, including plants, invertebrates and vertebrates, have a common and widespread geographic origin, and each has spread and colonized the region much earlier than previously thought. The results for the eight clades re-examined in this article suggest that they diversified and spread during the early Eocene, which helps to unify the geological and biological histories of IAA.

## 1. Introduction

The surface of our planet has been altered greatly by geological timescales, which has impacted the diversity of life at the highest level: many species are created and go extinct at the tempo of major geological events. Our ability to reconstruct the evolution of global biodiversity can therefore be achieved only by combining evidence from geology and biogeography. The Indo-Australian Archipelago (IAA) provides a prime example of this principle, because its striking biodiversity can only be understood by its geological dynamism [1,2]. The modelled geological history of IAA has critically influenced the biogeographic modelling of diverse clades (ranging from plants to vertebrates) and vice versa. However, new geological evidence has created a mismatch between biogeographic patterns and the connectivity of the landmasses in the region. Here, we aim to resolve this paradox by modelling and incorporating a key evolutionary process, species extinction, into biogeographic reconstructions.

The spatial configuration of islands and continental landmasses across IAA has changed considerably over geological timescales. There is a long-standing paradigm proposing that the Malay peninsula and Greater Sunda islands were totally disconnected from (at least) 60 [3] to 10 Ma, when the appearance of the islands now forming the Indonesian archipelago and Wallacea region could have served as stepping-stones for the dispersal of some clades. This geological hypothesis was supported by evolutionary studies conducted using modern geographic distributions and phylogenetic trees of extant species, which appeared to find constrained dispersal in ancient lineages across IAA, due to an extensive period when islands were not connected. Both animal [4] and plant [5,6] lineages that arose as early as 40 Ma underwent limited dispersal within but not between their centres of origin on either the Sunda or Sahul continental shelf, before dispersing elsewhere. Other taxonomic groups are documented to have originated in the Indochina peninsula, with further dispersal eastward to colonize New Guinea [7,8], while The Philippines are thought to have been colonized relatively recently [9]. Other clades seem to have originated in the eastern part of the region, followed by subsequent colonization events towards continental Asia [10]. Consistent with the idea of limited dispersal across the archipelago, widespread species are likely to form new species that become endemic to individual islands [11]. The fundamental role of this mechanism is reflected in high rates of vicariance (i.e. speciation due to differentiation between two populations with different islands). Under this view, dispersal facilitation by the late appearance of island stepping-stones is common to the biogeographical reconstructions of all the lineages that have been examined in recent studies [7,9].

Many studies have applied standard biogeographic reconstruction methods and weaved biogeographical hypotheses that are consistent with this geological hypothesis. However, this understanding of the geological history of IAA has recently been challenged by geomorphological evidence pointing to the presence of ancient land bridges between mainland Asia and the Indonesian islands [12–14]. For instance, it is hypothesized that Sundaland (i.e. the western part of the archipelago) was permanently continental until at least 6 Ma [12], which predates the onset of regional connectivity by tens of millions of years [15]. This hypothesis conflicts with the patterns seen in current reconstructions of biogeography for the region [4,7,16]. There is thus a mismatch between interpretations of the region’s history from the perspectives of geological and biological evidence.

One potential explanation for this disparity is that inferring the true biogeographic histories of clades is complicated by unrecorded species extinctions. Extinction inevitably removes the evidence of geographic distributions of extinct species in reconstructed phylogenetic trees. Traditional biogeographic approaches model only *local* extinction (also known as extirpation), which is different from lineage/species extinction. Recent work has demonstrated that two clades with the same history of speciation and rates of range evolution (i.e. colonization and local extirpation) can be inferred erroneously to have different origins and historical biogeographical dispersal events if they differ in background rates of lineage extinction [17,18]. Thus, radically different biogeographical reconstructions of regional biotas can be inferred when extinct lineages and their distributions are modelled explicitly. The amount of historical extinction in IAA is unknown but is likely to be high [19]. A discordance between current geological understanding of the region’s history and our best biological understanding might arise because lineage extinctions have not been accounted for in previous biogeographic inferences.

Specifically, we hypothesize that clades have used land bridges connecting the major IAA landmasses since their origin. If that is the case, we expect to find evidence against limitations in ancient dispersal, wider ancient geographic distributions and early colonization of the entire archipelago (compared to zero-extinction models). Furthermore, we expect that high connectivity among landmasses limits geographic isolation, which in turn decreases the likelihood of differentiation among allopatric populations (i.e. inhabiting different islands), leading to vicariant speciation, compared with *in situ* speciation (i.e. speciation within an island or location through any potential mechanism including fine-scale geographic isolation).

To test this hypothesis, we revisited the biogeographic history of clades that had been used previously to characterize the patterns of speciation and dispersal in IAA. These clades differ in their dispersal capacities and life histories and possess high-quality phylogenetic trees and well-known geographic distributions. We collated data used to reconstruct the phylogenies and biogeographic histories of eight clades representing plants, invertebrates and vertebrates (the breadfruits *Artocarpus*, orchids *Paphiopedilum*, treelets *Pseuduvaria*, taros *Alocasia*, crabs *Parathelphusa*, crickets *Cardiodactylus*, parachuting frogs *Rhacophorus* and herbs *Cyrtandra*) that have diversified across IAA over the last 45 million years. We considered the influence of extinction on the biogeographic reconstructions by explicitly modelling both the missing branches (due to extinction) on a phylogenetic tree and the geographic distribution of those extinct lineages. Our approach uses contemporary species distributions and does not require palaeogeographic information. We applied a likelihood framework where tree branches are used to compute the change in probability of a lineage (extant) being present at a given location. This probability also considers that a lineage could have existed at any point along a branch, could have changed across locations and went extinct before the present.

## 2. Results

**Figure 1.**
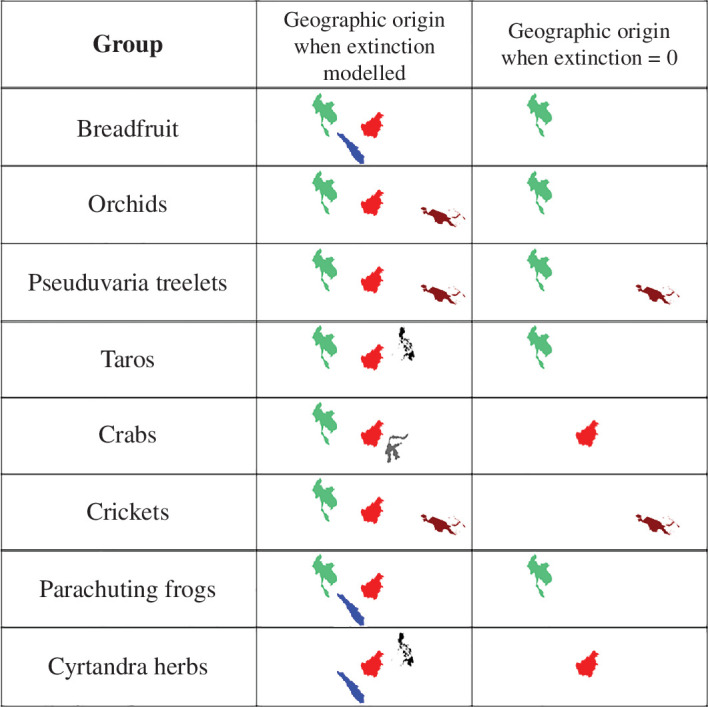
Geographic origins of eight clades in the IAA. For each dataset, we reconstruct the geographic distribution of the clade’s common ancestor while assuming intermediate and zero rates of lineage extinction.

**Figure 2.**
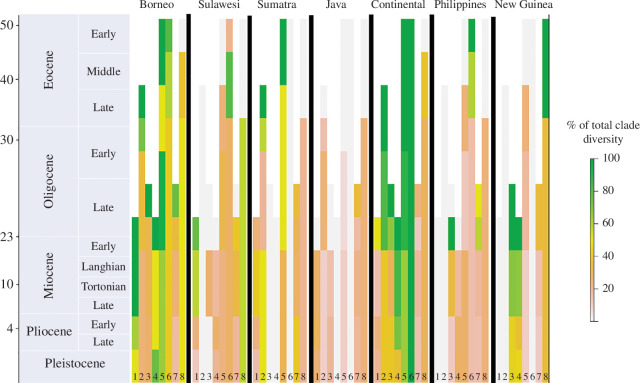
Reconstructed species richness over time across the IAA under intermediate rates of extinction scenario for eight taxonomic groups: (1) crabs, (2) parachuting frogs, (3) *Pseuduvaria* treelets, (4) orchids, (5) breadfruit, (6) taros, (7) *Cyrtandra* herbs, and (8) crickets. Colour code shows the relative number of species inhabiting each location at each time point. Notice that widespread ancestors contribute to the species richness of several locations. Time scale on the left is in millions of years. Similar figures but assuming low and high rates of extinction can be found in the electronic supplementary material.

**Figure 3.**
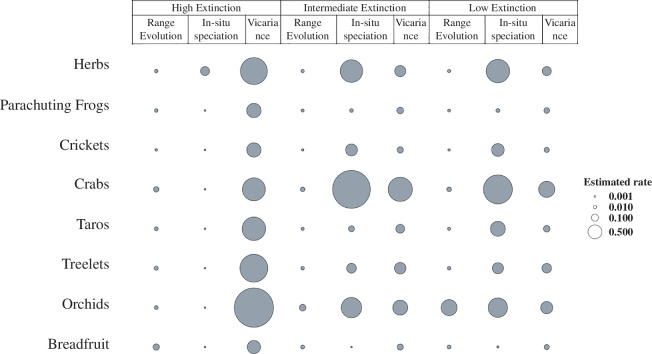
Rates of range evolution (colonization and extirpation), *in situ* speciation and vicariant speciation estimated during the reconstruction of ancestral geographic distribution for eight clades. For each dataset, we modelled three different scenarios that assume low, intermediate and high rates of lineage extinction.

By explicitly accounting for lineage extinctions, we obtain substantially different geographic origins and patterns of species distributions on the biogeographical histories of clades in the IAA from that inferred from models assuming zero extinction. When extinction is included, we find much greater concordance between the clades’ geographic origins (figure 1), we infer much earlier spread across the region for all clades (figure 2) and we find that *in situ* speciation becomes more important relative to vicariant speciation in generating the contemporary biodiversity of the region (figure 3).

### (a) A cradle for clades

When we set our models to assume zero extinction, we recovered the same geographic origins as those reported in the original studies. Models with zero extinction showed that all eight clades had narrow geographic origins that, in general, included either continental Asia or New Guinea. We found that the inferred geographic origin of the common ancestor of a clade changed when extinction rates higher than zero were assumed in the models. For instance, in *Cardiodactylus* crickets, a model assuming zero extinction reconstructs New Guinea as the original ancestral range, but when extinction was allowed, Borneo and continental Asia were inferred to be the centre of origin. In all cases apart from *Cyrtandra* herbs, we found that Borneo and continental Asia were included within the centre of origin when extinction was explicitly incorporated into models (figure 1).

### (b) Dispersal and early land connectivity in Indo-Australian Archipelago

We estimated substantially earlier dates at which clades arrived on different landmasses when models assumed lineage extinction rates higher than zero than when extinction was neglected (figure 1). For example, the breadfruit (*Artocarpus*) clade is predicated to have colonized The Philippines earlier in its evolution (22 Mya versus 10 Mya as previously estimated; figure 1); for taros (*Alocasia*), Sulawesi is estimated to have been colonized much earlier in models assuming intermediate/high extinction rates (35 Ma) than in models with zero extinction (7 Ma). For *Cytandra* herbs, the expansion out of Borneo is estimated to have occurred at least 2 million years earlier than previously thought; Sumatra, for instance, is estimated to have been part of the geographic range of *Cyrtandra* for the last 10.5 million years, whereas models assuming no extinction infer colonization of Sumatra only 6 Mya. Sulawesi is the location that shows more differences.

### (c) Mechanisms of speciation and species accumulation

Our revised models incorporating non-zero extinction reconstruct different patterns in the distribution of species diversity across IAA over time from zero-extinction models. For instance, The Philippines are estimated to have accumulated breadfruit species (*Artocarpus*) soon after colonization by the genus in the late Eocene (figure 2). Another interesting finding is that Borneo was richer in *Paphiopedilum* orchid species at 20 Mya than it is at present. Because our models assume a constant rate of extirpation over time, the reduction in orchid species in Borneo is not associated with an increased rate of extirpation. Instead, the decreasing orchid richness is caused by (global) processes that led to lineage extinction [20]. The low extinction scenario reconstructs Sulawesi to have low species richness through time (figure 2), in contrast with the high extinction case where Sulawesi harbours high diversity since the Eocene (electronic supplementary material, figures S25 and S26). Sulawesi is the location that shows more striking differences in how diversity accumulated over time across the different scenarios of species extinction. Taken together, these new reconstructions of the biogeographic histories of the eight clades provide a substantially altered picture of much earlier accumulation of species diversity and richness across IAA and much less certainty on the inference that these taxa had spatially disparate origins (figure 2; electronic supplementary material, figures S1–S24).

We find that the relative contribution of vicariance and *in situ* speciation also depends on the assumed extinction rate, and that this contribution varies across taxonomic groups. The contribution of *in situ* speciation increases with the assumed rate of lineage extinction. When lineage extinction is assumed to be low, vicariance is estimated to be higher than *in situ* speciation, except in breadfruits and parachuting frogs, where *in situ* speciation is the main mechanism of speciation. By increasing extinction to a more realistic intermediate rate, our analysis shows that *in situ* speciation also dominates for *Pseuduvaria* treelets and taros (figure 3). For the models assuming high rates of extinction, *in situ* speciation is the main mechanism behind diversification in all groups. When varying the assumed rates of extinction, the model does not compensate for changes in ancestral distributions by fitting high rates of range evolution. Instead, the estimated rates of range evolution are similar across different extinction rates, which demonstrates that our model successfully disentangles species extinction from extirpation (i.e. local extinctions).

## 3. Discussion

Our results suggest that the eight biological radiations in the Indonesian Archipelago we analysed are characterized by early and widespread dispersal, which results in a reconstructed widespread distribution of the common ancestor. We found that all clades were present within the landmasses represented by all the modern islands far earlier than was previously thought, and that expansion across the region generally occurred soon after the rise of a taxonomic group. As we included groups with large differences in dispersal capacities and evolutionary age, our results suggest that the movement of species throughout the region was not strongly constrained and that a large part of IAA might have been connected by islands or island-like land bridges for an extended period over the past 45 Myr. Importantly, it was necessary to account for species extinctions during lineage evolution to uncover these patterns. Under this scenario, vicariance processes are less likely to explain diversification, but, instead, speciation events take place within the areas that now form the major islands of contemporary Southeast Asia.

By modelling extinction, we found that the ancient dispersal of lineages across IAA took place much earlier than estimated by previous studies. Even though our conclusions are based on eight clades, they are congruent with recent geomorphological information [12] and fossil evidence. Fossil ostracods (Crustacea) have been found in the region of modern Java, and importantly, their subtidal lifestyle (based on eye tubercle morphology) suggests that land was available around 40–38 Ma [21]. The palynological record shows that mangroves were already inhabiting Sumatra during the Middle Eocene (~40–45 Ma [22,23]). Because mangroves occur at the interface between terrestrial and marine environments, this fossilized pollen also provides evidence of the presence of landmasses in the region at this time. These findings are incongruent with zero-extinction biogeographic reconstructions that typically conclude more recent colonization across the region, and they suggest that elevated rates of background extinction are required for accurate biogeographical reconstruction of the region. This conclusion is supported by climatic factors suggesting that lineage extinction is likely to be high in this region [19].

Our models consistently selected Borneo and continental Asia (plus New Guinea and Sumatra for three groups, The Philippines for two and Sulawesi one) as the geographic origin of the eight clades. The modelled taxonomic groups do not only differ in life history traits but also greatly vary in their evolutionary age, which suggests that the former landmass represented by these territories has consistently played an important role in shaping the biota of IAA [24]. Sundaland has been the cradle of entire taxonomic groups and also the stage of many speciation events, reinforcing its role as an evolutionary source of biodiversity rather than the destination [24,25]. For instance, the reconstructed common ancestor of *Cardiodactylus* crickets, which was previously thought to be of Sahul origin, is now shown to be present in Sundaland. When intermediate and high rates of extinction were assumed, our models suggest that continental Asia and Borneo have been occupied by all eight clades throughout their history.

Our results suggest that the estimate of the relative contributions of *in situ* and vicariant speciation changes when varying the assumed extinction rate. Models featuring high extinction rates estimate that, on average, *in situ* speciation is higher than vicariant speciation. There are two potential reasons for this. On the one hand, vicariance can take place only when species are geographically widespread, i.e. species present in more than one region. If extinction rates are high, many lineages are likely to disappear soon after they arise and inevitably before they have time to expand their geographic range. Accordingly, rates of vicariance are estimated to be relatively low in models that incorporate lineage extinction because these scenarios diminish the likelihood that widespread lineages arise and decrease the opportunities for vicariant speciation. On the other hand, during an *in situ* speciation event, the tally of local diversity increases by one species, which would then lower the probability that high extinction eliminates all species. For example, consider a scenario where lineage X occurring at areas A and B (*in situ*) speciates to produce lineage Y in area A, while the parent lineage X remains present across both A and B. Even if extinction removes lineage X entirely, area A remains occupied by lineage Y. Therefore, models with high rates of species extinction will be associated with high rates of *in situ* speciation, as this results in areas that are unlikely to become devoid of all species. If extinction is ignored, this process would appear to represent a range contraction of lineage X, which, according to our estimates, takes place at a low rate.

Our modelling approach simplifies the macroevolutionary dynamics taking place in the region. On the one hand, we assumed that diversification rates were uniform over time when in reality there are global, climate-related events that might have increased or decreased the rates of diversification. For instance, changes in atmospheric carbon dioxide concentration during Miocene have affected the radiation of many taxa on a global scale [26–28]. On the other hand, we assumed that diversification rates are uniform in space when, in fact, a combination of biotic and abiotic processes could have resulted in higher speciation rates in some locations than others. Similarly, lineages might experience increased probabilities of extinction in one area over another, although it would be truly challenging to detect such a signal from data as molecular phylogenetic trees are seldom informative on clade-wide extinction rates [29,30] and even less suitable for detecting differences in extinction rates between lineages. Although we recognize the potential importance of these patterns, they are not currently represented in our models, which aim to reconstruct the biogeographic history of the region when assuming different extinction rates, but do not estimate extinction rates from phylogenetic trees.

Integrating extinction dynamics into ancestral reconstructions is crucial for reconciling evolutionary processes shaping the modern patterns of species diversity with geological and palaeontological evidence. A similar re-evaluation of lineage evolution incorporating non-zero rates of extinction was required to reconcile the contemporary biogeography of hummingbirds with the fossil record and demonstrated that the common ancestor of hummingbird species lived in North America [17] rather than South America [31,32]. Our results encourage the use of interdisciplinary and complementary approaches to address questions that cannot, otherwise, be addressed comprehensively.

## 4. Methods

We collated published papers that reconstructed the biogeographic history of clades in IAA and selected those whose geographic range had limited departure from the following geographic localities: Borneo, Sulawesi, Sumatra, Java, Philippines, New Guinea and continental Southeast Asia. The taxonomic scope of these studies included plants and both invertebrate and vertebrate animals: breadfruit (*Artocarpus* [6]), orchids (*Paphiopedilum* [33]), treelets (*Pseuduvaria* [16]), taros (*Alocasia* [5]), crabs (*Parathelphusa* [34]), crickets (*Cardiodactylus* [4]), parachuting frogs (*Rhacophorus* [35]), and herbs (*Cyrtandra* [7]). All those studies applied either dispersal-extinction cladogenesis (DEC) [36] or dispersal-vicariance analysis (DIVA) [37] models for biogeographic reconstruction. The authors kindly provided phylogenetic trees and geographic information used for their analyses.

We used the modelling framework Lineage Extinction Model of Ancestral Distribution (LEMAD) to revisit the geographic distribution of the ancestors in these groups. Unlike previous methods, LEMAD explicitly models the distribution of extinct lineages in geographic reconstruction [17]. DEC and similar approaches use the set of extant species to reconstruct past changes in geographic distributions, while LEMAD includes extant species but also models extinct species (see below) to reconstruct the biogeographic history. The amount of historical extinction in IAA is unknown but is likely to be high [19], and models other than LEMAD neglect this key process. Notice that DEC and other approaches consider *local* extinction (also known as extirpation), which is the change from the presence to the absence of a lineage at a given location: a species/lineage could still exist in other locations and remain extant to the present. Radically different biogeographical reconstructions of regional biotas can be inferred when extinct lineages and their distributions are modelled explicitly [17]. The inference of lineage extinction (hereafter extinction) rates from molecular phylogenetic trees is challenging and might lead to the estimation of biased rates because of taxonomic sampling issues [38] and heterogeneity of rates across lineages [30]. LEMAD does not attempt to estimate extinction rates; instead, it is used to explore the reconstructed ancestral distributions when assuming different extinction rate values in order to address this important source of uncertainty.

LEMAD generalizes the likelihood described in GeoSSE [39] for any number of areas and is flexible to include different scenarios of geographic speciation that facilitate the estimation of ancestral distribution. Like GeoSSE, the change in geographic distribution of species is a result of species colonizing locations (dispersal) and becoming extirpated from locations (i.e. the disappearance from a local area, also known as range contraction or local extinction). Unlike GeoSSE, LEMAD assumes that rates of speciation and extinction are uniform across regions. Extinction is modelled as an instantaneous process across the entire range of a lineage, which allows us to account for those events where populations experience a sudden decline in size and are unlinked to geographic range contractions [39]. In LEMAD, both the phylogenetic tree and geographic information are jointly used to carry out the calculation. A system of equations is defined to represent (i) the probabilities of a given branch (i.e. an existing branch) being present at a different geographic location and (ii) the probabilities of a branch that, having existed at a different geographic location, went extinct. For instance, consider that lineage Z can have any of three distributions (area A, area B or being present in both A and B), LEMAD defines the probability of lineage Z being present in A coupled with an equation that reflects the possibility of an extinct lineage that was present in A before going extinct. The same computation is carried out for area B and the area represented by both A and B. The assumed extinction rate is defined by the user. The equations also include a term that accounts for changes in geographic distributions, i.e. lineages colonizing or disappearing from locations. These equations are numerically integrated along all the tree branches from the tree tips (using the geographic information of extant lineages) to the root. Once the likelihood is optimized, these probabilities are retrieved at each node along with the rate estimates for dispersal/extirpation, *in situ* speciation and vicariant speciation (geographically mediated divergence resulting in allopatry, i.e. complementary ranges).

Vicariant and *in situ* speciation can be modelled in two different ways. On the one hand, the DEC model [36] assumes that during vicariance, one of the daughter lineages will be present in only one region (e.g. the four species ABC and D are partitioned geographically into A-BCD or B-ACD; narrow vicariance); for *in situ* speciation, the DEC model allows that a population from a widespread species diverges to form a new species, which co-occurs with the parental one (i.e. *in situ* subset). On the other hand, the DIVA model [37] assumes that widespread species can split their ranges with no restriction in the number of areas inhabited by daughter lineages, as long as they form complementary distributions (e.g. a species present in regions A, B, C and D can split into AB-CD or A-BCD; widespread vicariance). In DIVA, the *in situ* subset mode is not assumed. In the LEMAD framework, DEC and DIVA are different versions of the same model (LEMAD_DIVA_ and LEMAD_DEC_); they differ in the arrangement of parameters; thus, their likelihoods are comparable. We fit LEMAD_DIVA_ and LEMAD_DEC_ to the revisited datasets. Because the current distribution of most species across revisited studies is restricted to one or two areas and to be in line with the original analyses, the maximum number of areas where ancestors could have inhabited was set to three. In the LEMAD analysis and in contrast to some of the original IAA studies, our models did not include a time-stratified component or jump dispersal.

For each dataset, we ran four models that differed in the assumed rates of extinction. The decision on what extinction rate to assume is not straightforward. In the field of macroevolution, estimates for extinction rates calculated from phylogenetic trees are generally small, often close to zero, which contradicts the fossil record [38]. We fitted a standard birth–death (BD) model to each phylogenetic tree and confirmed that the extinction rate was estimated to be close to zero. With a highly incomplete fossil record and no external evidence that could suggest a reliable rate of extinction for the revised datasets, we took an alternative approach. Instead of using those clearly underestimated extinction rates from a BD model, we assumed that extinction could have been almost as frequent as speciation, as shown in datasets with the most complete fossil records [40,41]. We therefore used the speciation rate estimate under a BD model for each dataset and termed this rate BD_mu. This was the assumed extinction rate for the first model. In the second model, we assumed a much higher rate of extinction (10 × BD_mu) [42]. The third model assumed low extinction (BD_mu/10). It is reasonable to assume that the extinction rate adopted for the second and third models brackets the actual range of values for each lineage, while that adopted in the first model is a tentative estimate of its long-term mean. Finally, we fitted models assuming zero extinction. Note that during LEMAD likelihood optimization, speciation and range evolution rates are adjusted according to the assumed extinction rate (i.e. the speciation rate is in all cases higher than extinction). We allowed the rates of *in situ* and vicariant speciation and range evolution (i.e. colonization and local extinction hereafter extirpation) to be free parameters in the model. We found that LEMAD_DEC_ models had better likelihood than LEMAD_DIVA_, so we report the results of the former. All analyses were carried out using one phylogenetic tree per clade that was provided by the authors of the original papers.

## Data Availability

Code to perform the biogeographic analysis and two of the revisited datasets (granted permission by the original authors to make datasets public) are available at Dryad [43]. Supplementary material is available online [44].
